# Plasminogen activator activity in tears of pregnant women

**DOI:** 10.1371/journal.pone.0177003

**Published:** 2017-05-04

**Authors:** Adrienne Csutak, Zita Steiber, József Tőzsér, Attila Jakab, András Berta, David M. Silver

**Affiliations:** 1 Department of Ophthalmology, Faculty of Medicine, University of Debrecen, Debrecen, Hungary; 2 Department of Biochemistry & Molecular Biology, Faculty of Medicine, University of Debrecen, Debrecen, Hungary; 3 Department of Obstetrics and Gynecology, Faculty of Medicine, University of Debrecen, Debrecen, Hungary; 4 Johns Hopkins University, Bethesda, Maryland, United States of America; Save Sight Institute, AUSTRALIA

## Abstract

**Purpose:**

Plasminogen activator activity (PAA) in tears of pregnant women was investigated at various gestation times to assess the availability of plasminogen activator for aiding potential corneal wound healing processes during pregnancy.

**Methods:**

PAA was measured by a spectrophotometric method. The analysis used 91 tear samples from pregnant and non-pregnant women, supplemented with 10 additional tear PAA measurements from non-pregnant women obtained in a previous study.

**Results:**

Tear levels of PAA in pregnant women formed a bimodal distribution. Either the tear PAA level was zero or non-zero during pregnancy. When non-zero, the tear PAA level was dissociated from gestation time and not different than non-pregnant and post-pregnant levels. The frequency of occurrence of zero level tear PAA increased with gestation: 16%, 17% and 46% had zero tear PAA in samples taken from women in the first, second and third trimester, respectively.

**Conclusions:**

Overall, of the tear samples taken from women during pregnancy, a total of 26% were at zero tear PAA. The remaining tear samples had non-zero tear PAA values throughout gestation equivalent to non-pregnant tear PAA values, suggesting local control of the source of PAA in tears. Given the importance of the plasminogen activator system in tears to wound healing in the cornea, and the high occurrence of zero tear PAA in our sample of pregnant women, elective corneal surgery would be contraindicated. If corneal surgery is nevertheless necessary, the tear PAA level would be worth checking and patients with low level should be closely observed during the postoperative period.

## Introduction

Major changes in hemostasis are associated with normal pregnancies [1,2]. Thrombosis is rare in the first trimester of pregnancy but by the later trimesters both coagulation and fibrinolysis are enhanced, thus presenting normal pregnancy as a hypercoagulable state. The plasminogen activator/inhibitor system plays a key role. The specific serine protease plasminogen activators convert inactive plasminogen to active plasmin, thus promoting extracellular proteolysis [3]. The coagulation activity, the anticoagulation activity, fibrinolytic and anti-fibrinolytic activity are enhanced in normal pregnancy but these activities are balanced despite large changes in blood levels of plasminogen activators and inhibitors [4,5]. This balance and change are needed to control the delivery environment [6,7]. The systemic proteolytic activity needs to be kept under sensitive control to avoid serious complications that may accompany a disturbance of this system [8,9].

Two types of plasminogen activator exist, tissue-type plasminogen activator (tPA) and urokinase-type plasminogen activator (uPA). The plasminogen activator cascade is controlled at different levels, one of which is the inhibition of plasminogen activator activity (PAA) by plasminogen activator inhibitors (PAIs). The first of the two types of PAIs is the plasminogen activator inhibitor type-1 (PAI-1), which is produced by vascular smooth muscle cells, platelets and hepatocytes. The second type of PAI is the plasminogen activator inhibitor type-2 (PAI-2), which is mainly produced by trophoblasts and this plays an important role in inhibiting uPA during pregnancy [10].

Plasminogen activators and PAIs are found at elevated levels in the blood of pregnant women and in gestational tissues [11–14], During pregnancy, activity and antigen levels of uPA are found elevated in plasma [7,15,16], peaking around the third trimester, fall during labor, and return to normal non-pregnant levels post-partum [6].

In normal healthy eyes, uPA is a normal component of tear fluid that originates from conjunctival and corneal epithelial cells, while tPA is not detectable in tears [17]. PAI-2 is detectable in tears and may play a role in the inhibition of uPA [18], but levels of PAI-1 [19] are below the detection limit of 4 ng/ml of the ELISA test in normal non-pregnant patient’s tears.

In previous measurements on human tears, a characteristic pattern of PAA was observed before and after corrective laser surgeries, laser in situ keratomileusis (LASIK) and photorefractive keratectomy (PRK) [19]. The results showed an absence of PAI-1 but a measurable PAI-2 level present in tears prior to and after surgery [19]. An enzymatic control response to corneal surgical wounding was asserted. In contrast, low PAA sustained over a period of three days, evidenced in tear fluid, was an accompanying sign of corneal haze 3–6 months after the surgery in a human study [17].

In a rabbit study, uPA was inhibited in eight eyes with aprotinin, a serine protease inhibitor, and thereby induced haze [20]. Similar results were reported when a human patient was treated with aprotinin after PRK [21,22]. From these results, low PAA was hypothesized to be a possible cause of defective corneal wound healing. In another rabbit study, pregnancy was identified as a risk factor for the development of corneal haze after photorefractive laser treatment [23].

This raised a question about the level of PAI’s and PAA during pregnancy. Therefore further previous work measured levels of PAI-2 in the tears of pregnant women at different stages of pregnancy, finding that PAI-2 levels in tears of pregnant women were not different than the levels found in non-pregnant women and were not correlated with gestational age [24]. In the present work, we explore the levels of PAA in tears of pregnant women to assess the potential risk pregnant women may have for abnormal corneal wound healing.

## Materials and methods

### Subjects

Tear samples were selected for this study after obtaining written informed consent from the women in adherence to the guidelines of the Declaration of Helsinki. Ethical approval for this work was obtained from the University of Debrecen Ethics Committee (2155–2004). The women were tested opportunistically either prior to pregnancy, between 9 and 39 weeks of pregnancy, or within 1 week after delivery. Only two subjects returned for a second measurement at a later gestational time: these were considered independent measurements. The subjects were all healthy women with normal pregnancy, without pre- or postnatal difficulties. None of the subjects had a history of any general disease, familial disorders, hemostatic disorders, or other relevant diseases or taking of any drugs.

Before the tear sampling, the women underwent routine eye examinations, with the anterior segment examined under slit lamp. All women had normal corneal surface, good visual acuity (VA 1,0; with or without correction), no previous eye injury or surgery, no history of any eye disease (nor in the family), no history of any general medication or topical use of eye drops, and, during the examination of these women, no occurrence of corneal injury or incision. Contact lens wearers were excluded from the study cohort.

The number of women and their ages in each gestational category is tabulated in Table 1.

**Table 1:** Age (years) and number of participants in each category.

	Non-Pregnant	9 to 12 Weeks	14 to 23 Weeks	25 to 39 Weeks	First Postnatal Week
Mean Age	28.7	34.2	32.6	30.1	32.1
SD Age	7.9	5.7	4.5	4.9	5.4
Maximum Age	46	42	39	42	39
Minimum Age	17	19	23	21	23
Number of participants	18	19	30	24	10

### Tear sampling

Tear samples were collected from the patients’ left eyes at the outpatient clinic of the Obstetrics and Gynecology Department, using the same, standardized collection method throughout the study. Samples consisted of tears collected with open-ended, 70 mm long, 1.2 mm diameter glass capillaries (VWR International LLC, Radnor, PA, USA) from the lower tear meniscus (a horizontal thickening of the pre-corneal tear film by the lower margin) at the lateral canthus without touching the conjunctiva. The duration of the sampling times was less than 2 minutes, without stimulation [25] to avoid dilution from reflex tearing [26,27], Sampling times were recorded and the secretion rates were calculated in μl/min (5–15 μl/min). Samples were centrifuged (1800 rpm) for 8–10 minutes right after sample collection and supernatants were deep-frozen at -80°C and were thawed only once for measurements.

### PAA measurements

PAA was measured in the sample tears by a spectrophotometric method using human plasminogen and a plasmin-specific chromogenic peptide substrate, D-valyl-L-leucyl-L-lysine-p-nitroanilide (S-2251) [28]. This assay is sensitive predominantly to urokinase plasminogen activator [28]. Plasminogen was purchased from Abcam (Cambridge, MA, USA) and the S-2251 was purchased from Chromogenix (Môlndal, Sweden). Urokinase standard was purchased from Choay (Paris, France). This assay is suitable to measure plasmin activity but can also be used for determining plasminogen activator activity by adding plasminogen to the reagents. Plasminogen activator activity was measured as described by Shimada and coworkers [24] with the following modifications according to Tőzsér and coworkers [29,30]: 5 μl tear, or standard urokinase, or plasmin was incubated in 100 μl of 0.05 Tris buffer, pH 7.4, at 37°C in the presence of 0.5 mmol/l chromogenic substrate S-2251 and 1 μmol/l human plasminogen in wells of microtiter plates. After 4 hours incubation, the reaction was terminated by the addition of 100 μl of 8 mol/l acetic acid. The absorption was measured at 405 nm with a Wallac VICTOR2 1420 fluorimeter-luminometer (Wallac Oy, Turku, Finland). Plasminogen independent amidolytic activity was measured similarly but plasminogen was omitted from the incubation mixture. The absorption difference between the values obtained with and without plasminogen was considered to be due to the plasminogen activator activity in tear, while the absorbance value obtained without plasminogen was considered as plasminogen independent amidolytic activity. A calibration curve was produced based on the absorption values gained in the same system with different concentrations of urokinase standard solutions. The plasminogen activator activities of the measured samples were calculated with this calibration curve and were expressed in IU/ml urokinase equivalent values.

The tear sample measurements from women prior to pregnancy were used as a control to compare with the tear sample measurements made on pregnant women. Due to the small number of subjects presenting at the Obstetric Clinic before pregnancy, 10 additional PAA measurements were added from non-pregnant women, who participated in a previous study [17] conducted at the Department of Ophthalmology, Faculty of Medicine, University of Debrecen. The tear collection, PAA measurement procedure, and analysis used for the previous study were the same as used in the present study. The current and previous non-pregnant tear measurements were subsequently compared for equivalence.

### Statistical methods

A total of 104 tear measurements were considered in this work and were tested against the Grubbs criteria for outliers [31], leaving 101 measurements for further analysis, S1 Table. The PAA values were then divided into gestational categories as follows: non-pregnant (18 samples), 9 to 12 weeks (19 samples), 14 to 23 weeks (30 samples), 25 to 39 weeks (24 samples), and 1^st^ postnatal week (10 samples). Tear levels of PAA were plotted against these gestational categories. Normal distribution of samples within each gestational category was tested with analysis of residuals from a normal plot [32]. Equivalence of PAA levels across gestational categories were tested with t-tests, median tests using a chi-squared distribution with Yates correction for continuity, Mann-Whitney U tests and one-way analysis of variance [33]. Statistical significance was defined as p ≤ 0.05.

## Results

The PAA measurements in the non-pregnant category came from two sources of measurement: 10 from previously tested non-pregnant women and 8 current women tested prior to pregnancy. Using t-tests, there was no statistically significant difference from the previous and the current non-pregnant mean PAA values (p = 0.63) nor ages of the participants (p = 0.26). The Mann-Whitney U tests showed no statistically significant difference from the previous and current non-pregnant PAA median values (p > 0.99). The median test using a chi-squared distribution with Yates correction for continuity showed no significant difference in PAA values between the two groups (p = 0.64). Accordingly, in subsequent analyses, the 18 PAA measurements were considered equivalent for further analysis.

**Fig 1:**
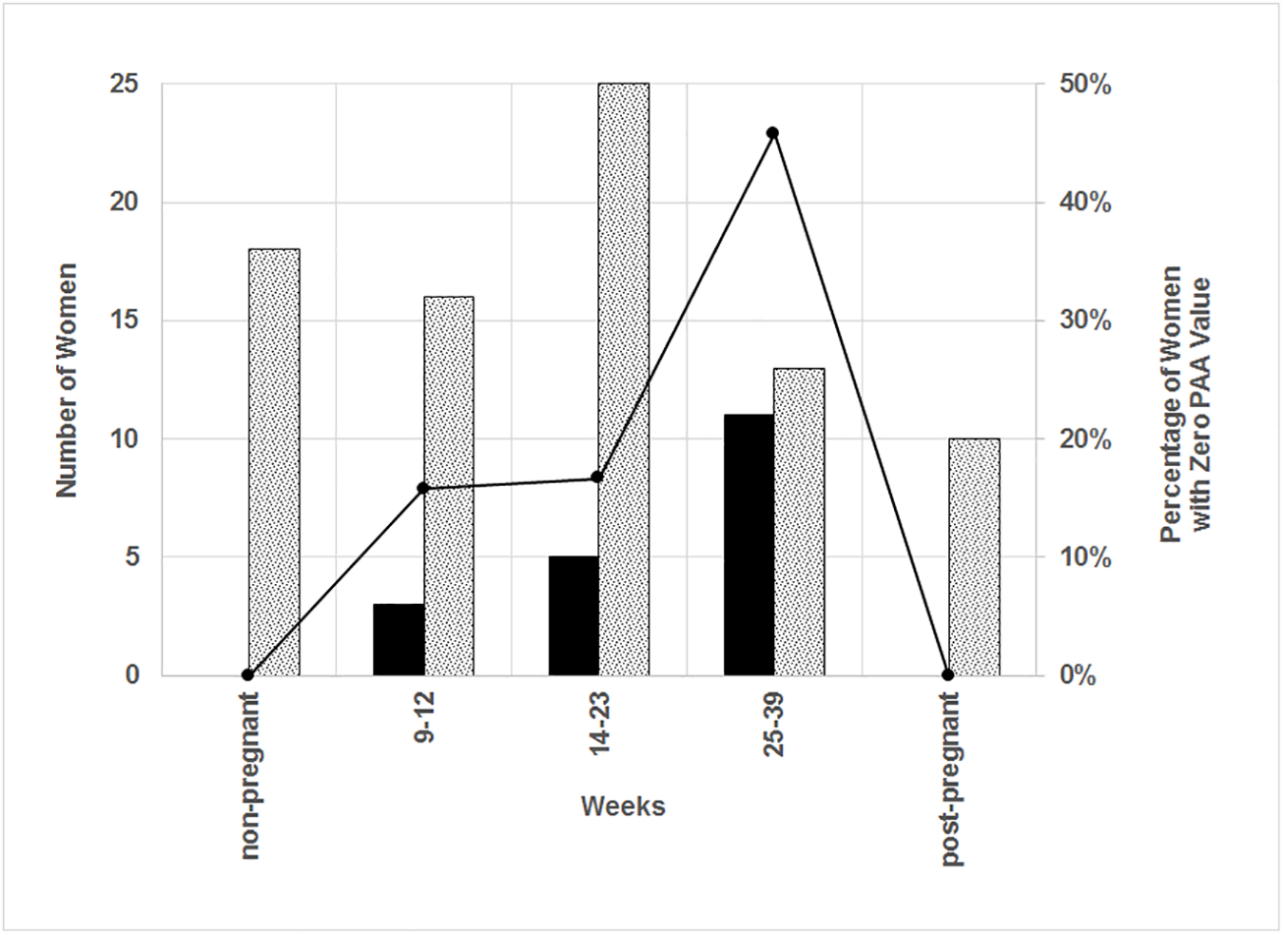
Number of plasminogen activator activity measurements versus gestation. Shaded bars represent the number of PAA measurements with non-zero values and solid bars represent the number of PAA measurements with zero value (left scale). The solid line represents the percentage of PAA measurements with zero value (right scale).

The number of tear sample measurements is shown in Fig 1 plotted against the gestational category. In particular, the numbers of samples where the PAA value was zero are shown as solid bars. The percentage of samples having zero PAA value compared to the total number of samples at a particular gestation category is also shown. The percentage of zero PAA increases with increasing gestational category;16%, 17% and 46% of the samples had zero PAA in the first, second and third trimester, respectively, but none of the non-pregnant or post-pregnant PAA samples had zero PAA values. In addition, in a previous study [17] on non-pregnant healthy individuals, using the same tear collection method as used here, PAA measurements were non-zero, including some that showed low but detectable PAA.

Because of the bimodal distribution of PAA values, the results were separated into two groups: non-zero PAA and zero PAA for three of the gestational categories. The number of samples and PAA results in each of the categories is given in Table 2.

**Table 2:** PAA (IU/ml) and number of participants in each category.

	Non-Pregnant	9 to 12 Weeks	14 to 23 Weeks	25 to 39 Weeks	First Postnatal Week
Mean Non-Zero PAA	0.325	0.243	0.309	0.348	0.339
SD Non-Zero PAA	0.184	0.129	0.180	0.228	0.217
Maximum Non-Zero PAA	0.785	0.504	0.780	0.809	0.831
Minimum Non-Zero PAA	0.050	0.005	0.076	0.046	0.191
Number (non-Zero PAA)	18	16	25	13	10
Number (zero PAA)	0	3	5	11	0

The age distribution of the women in the various categories was tested with Mann-Whitney U tests, which indicated that there were no statistically significant differences in the ages of the participants between non-zero PAA and zero PAA within each of the three gestational categories: p = 0.96, 0.56 and 0.95, respectively. Median tests of the ages of participants between non-zero PAA and zero PAA within each of the three gestational categories showed no statistically significant differences: p = 0.61, 0.87 and 0.71, respectively. Across all five of the gestation categories (combining non-zero PAA and zero PAA), variance of ages within a gestation period was 31.8 while variance of ages between gestational categories was 72.0, giving a variance ratio of 0.44 with p = 0.95. Therefore, most variance in age is between gestational categories, not in the ages of women within the gestational categories. Tests for normal distribution of ages for the five gestational categories showed a linear fit of the normal plot residuals: each of the five R^2^ > 0.85.

**Fig 2:**
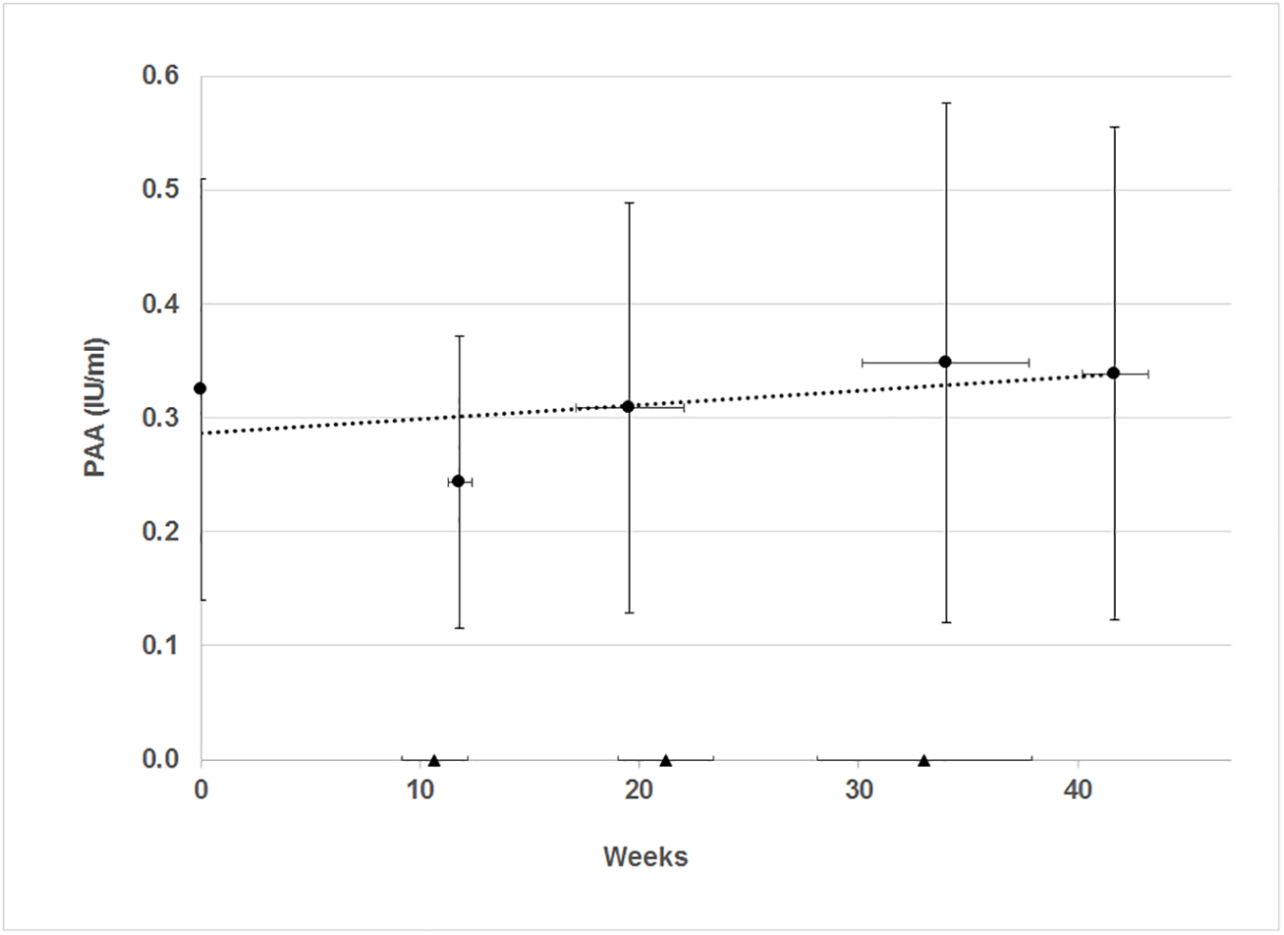
Plasminogen activator activity versus gestation. Circles represent PAA measurements with non-zero values and triangles represent PAA measurements with zero value. Vertical error bars represent standard deviations of PAA about the mean values. Horizontal bars represent standard deviations about the mean position of weeks. Dotted line represents the trend of the non-zero PAA values (Y = 0.0012 X + 0.29 and R^2^ = 0.25).

The PAA results are shown in Fig 2 against gestation category. The tests for normal distribution of the non-zero PAA measurements within each gestation category showed reasonable normal distribution: each R^2^ > 0.61, corresponding to a linear fit of the normal plot residuals.

Using t-tests, there is no statistically significant difference between the mean PAA value at each of the five gestation periods shown in Fig 2, p > 0.13. The median test confirmed equal medians across the gestational age categories, p = 0.79. The Mann-Whitney U tests showed no statistically significant difference between the median of the PAA values at each of the five gestation periods compared with one another, p > 0.18. Further, the analysis of variance shows PAA variance = 0.034 within the five gestation times and PAA variance = 0.020 between the five gestation periods, giving a variance ratio of 1.69 with p = 0.29. Thus, most variance is within the participants’ PAA measurements taken in the same gestation period, not between PAA measurements taken at different gestation times.

The mean non-zero PAA levels remained essentially unchanged across categories from non-pregnant, through pregnancy and post-pregnant. However, the number of zero PAA values increased with gestation age category, but before and after pregnancy, no zero PAA level measurements were seen.

One of the two women from whom tear samples were collected at two gestation categories had a non-zero PAA value (0.427 IU/ml) before pregnancy and a zero value when measured at 9 weeks. The other woman had non-zero PAA values in both samples.

## Discussion

Of the 73 measurements of PAA in tears of women who were pregnant (9 to 39 weeks) at the time of measurement, 26% had zero levels of PAA with the fraction of women with zero PAA increasing with later gestational age. The remaining 74% of PAA measurements in tears of pregnant women were non-zero and those PAA levels were equivalent to the levels measured from non-pregnant, pre-pregnant and post pregnant women.

Plasminogen activators, measured in blood samples taken from pregnant women, show a definite dependence on the progression of pregnancy, increasing with gestation and returning to normal levels post-partum [6,11,14–16]. In contrast, the non-zero PAA values reported here from tears are independent of the progression of pregnancy and not influenced by changes in systemic variations. This suggests the possibility of local control of the source of uPA in tears. A similar conclusion was drawn with respect to PAI-2 from tears [24], where the tear-derived PAI-2 levels were found to be independent of gestation, while PAI-2 from blood samples had a dependence on gestation time.

Both plasminogen activators, uPA and tPA, and plasminogen activator inhibitors, PAI-1 and PAI-2, are found in gingival crevicular fluid (GCF) from both male and female subjects at levels all more than ten times higher than in term pregnancy plasma [34]. In GCF, the concentration of tPA is 10–15 times higher than uPA, while the concentration of PAI-2 is at least 5 times higher than PAI-1 [34]. Because of these ratios, the focus of attention is on tPA and PAI-2 in GCF fluid [35,36]. Often but not always during pregnancy, a more vigorous gingival inflammatory response to bacterial plaque is seen [37]. However, concentrations of the dominant species, t-PA and PAI-2, in GCF are not significantly different between levels measured in GCF during pregnancy and post-partum [38]. The observation is that the plasminogen active substances in GCF are not affected by pregnancy. The implication is that they are locally produced in the gingiva, not controlled by their concentration levels in blood [38,39].

Various biological processes including cell adhesion, cell migration, and tissue remodeling depend on the presence of uPA. For instance, an up-regulation of uPA in corneal epithelial cells occurs after mechanical wounding of the cornea, contributing to epithelial cell migration [40]. For another example, allergic conjunctivitis is accompanied by elevated uPA levels in tears and conjunctival epithelial cells, where the uPA is involved with proteolysis and cell adhesion, cell migration and tissue remodeling [41]. These results illustrate the interlinkage between fibrinolytic machinery and inflammatory activity [42]. What would occur if corneal wounding or conjunctivitis happened to a pregnant woman whose uPA level is zero during pregnancy?

Previous work found that an absence of PAA in tears after laser vision correction surgery correlated with a tendency to incur an incomplete corneal wound healing experience [17,20,23] and that pregnancy was a risk factor for developing corneal haze after PRK [23]. Pregnancy can be accompanied by changes in refraction and other corneal properties [43,44]. Eyes of women who became pregnant after PRK have experienced refractive regression and corneal haze [45]. Changes in visual acuity and spherical equivalent have been reported in eyes of women who became pregnant after LASIK [46]. Although pregnancy normally precludes corneal laser surgery [47], sometimes an early stage pregnancy has inadvertently not been identified. Also, traumatic corneal injury may require corneal surgery or other procedures involving wound healing.

With the increase in prevalence of myopia worldwide [48–51], especially among younger people, LASIK and PRK are procedures often chosen for amelioration. Thus corneal wound healing becomes increasingly important, including a need to be sensitive to changes in tear enzymes that affect the corneal wound healing process. The level or absence of enzymes in tears during pregnancy should be an important consideration when corneal surgery on a pregnant woman is contemplated.

## Supporting information

S1 Table PAA in tears of pregnant women: Analysis data. (XLSX)
